# Current biological and pharmacological updates on wogonin

**Published:** 2020-05-13

**Authors:** Sarita Rawat, Gaurav Gupta, Sachchidanand Pathak, Santosh Kumar Singh, Himmat Singh, Anurag Mishra, Ritu Gilhotra, Alaa A. A. Aljabali, Harish Dureja, Murtaza M. Tambuwala, Dinesh K. Chellappan, Kamal Dua

**Affiliations:** 1 School of Pharmacy, Suresh Gyan Vihar University, Mahal Road, Jagatpura, Jaipur, India; 2 Faculty of Pharmacy, Department of Pharmaceutics and Pharmaceutical Technology, Yarmouk University, Irbid 21163, Jordan; 3 Department of Pharmaceutical Sciences, Maharshi Dayanand University, Rohtak, Haryana 124001, India; 4 School of Pharmacy and Pharmaceutical Sciences, Ulster University, Coleraine, County, Londonderry, BT52 1SA, Northern Ireland, United Kingdom; 5 Department of Life Sciences, School of Pharmacy, International Medical University, Bukit Jalil, Kuala Lumpur, Malaysia 57000; 6 Discipline of Pharmacy, Graduate School of Health, University of Technology Sydney (UTS), Ultimo, NSW 2007, Australia; 7 Centre for Inflammation, Centenary Institute, Sydney, NSW 2050, Australia; 8 Priority Research Centre for Healthy Lungs, Hunter Medical Research Institute (HMRI) & School of Biomedical Sciences and Pharmacy, The University of Newcastle (UoN), Callaghan, NSW 2308, Australia

## ⁯

***Dear Editor,***

Wogonin (5, 7-Dihydroxy-8-methoxy flavone) is a traditional naturally occurring flavonoid derived from the root extract of Chinese medicine, named *Scutellaria baicalensis *Georgi. Wogonin contains various biological properties which include allergic diseases, anti-cancer therapy, and anti-inflammatory activities. Wogonin also shows the effects of removing toxins and cleansing the heart (Ancuceanu et al., 2019[1]). The anticancer therapeutic activity of wogonin has been shown by the regulation of different cell signaling pathways, which includes protein kinase B pathway (serine-threonine kinase) and AMP-activated protein kinase pathways (Bei et al., 2020[2]). Wogonin also shows positive therapeutic anticancer effects in breast cancer by inhibiting the 5-LO/BLT2/ERK/IL-8/MMP-9 signaling cascade and establishes a major pharmacological anticancer activity (Bibi et al., 2019[3]). Current biological and pharmacological updates on wogonin have been reviewed (Table 1(Tab. 1); References in Table 1: Du et al., 2019[4]; Ewendt and Foller, 2019[5]; Fang et al., 2019[6]; Gao et al., 2019[7]; Gharari et al., 2020[8]; Hanioka et al., 2020[9]; Hong et al., 2020[10]; Huang et al., 2020[11]; Jiang et al., 2019[12]; Jiao et al., 2019[13]; Khan and Kamal, 2019[14][15]; Khushdil et al., 2019[16]; Kim et al., 2019[17]; Kong et al., 2019[18]; Liang et al., 2019[19]; Liau et al., 2019[20]; Luo et al., 2019[21]; Oomen et al., 2020[22]; Tan et al., 2019[23]; Wang and Cui, 2019[24]; Wang et al., 2019[25][26]; Wang et al., 2020[27]; Xing et al., 2019[28][29]; Zhang et al., 2019[30]).

| Key findings | Reference |
|---|---|
| In osteoarthritis, wogonin shows its therapeutic effects by inhibiting IL-1β-induced gene expression of MMP-1, MMP-13, and ADAMTS-4, and also restores the gene expression of type II collagen that has been inhibited by IL-1β. | Du et al., 2019 |
| Wogonin attaches to chondrocytes DNA through an intercalation mechanism. It was set up to be limited in the nucleus and exhibit protective effects in osteoarthritis chondrocytes by inducing the anti-apoptotic proteins. Wogonin suppresses the IL-1β mediated induction of ROS, DNA fragmentation and also extrinsic and intrinsic apoptotic pathways. | Ewendt and Foller, 2019 |
| Wogonin and its homologous compounds reserved the viabilities of human hepatoma cells accompanying the loss of MMP and the exhaustion of glutathione content preferentially induced apoptosis through the intrinsic pathways in human hepatoma (HepG2) cells by the measurement of the representative indexes of apoptosis and the control of overexpression of Bcl-2. On the other hand, wogonin is not involved in the growth of embryonic hepatic L02 cells at lower concentrations but induces slight apoptosis at high concentration (200 mM). It shows that wogonin preferentially kills tumor cells. | Fang et al., 2019 |
| The anticancer mechanism of wogonin has been recognized by modulation of various cell signaling pathways, which include serine-threonine kinase *Akt* (protein kinase B) and AMP-activated protein kinase (AMPK) pathways and inhibition of telomere activity. Also, wogonin decreases DNA adduct formation with a carcinogenic compound 2-Aminofluorene and inhibits the growth of drug-resistant malignant cells and their movement and metastasis, without any side effects. In recent times, newly synthesized wogonin derivatives have been identified with notable anti-tumor activity. | Gao et al., 2019 |
| In recent research, it was found that wogonin suppresses the LPS-enhanced invasiveness and metastasis of breast cancer cell lines by showing an inhibitory effect on the lipopolysaccharide (LPS)-enhanced invasiveness of MDA‑MB‑231 cells. Wogonin also inhibits the synthesis of matrix metallopeptidase‑9 (MMP‑9) and interleukin‑8 (IL‑8), which makes it critical to promote invasiveness in MDA‑MB‑231 cells. | Gharari et al., 2020 |
| Wogonin shows renoprotective effects for cisplatin-based anticancer therapy. In a recent study it was found that wogonin substantially decreased the increased levels of serum creatinine and blood urea nitrogen (BUN) nearly to the normal level. It effectively inhibited RIPK1 by occupying the ATP-binding pocket of the enzyme, which is a type of regulator of necroptosis. Shockingly, wogonin improved the antiproliferative outcome of cisplatin on human hepatoma HepG2 cells. As a result, wogonin may be used in cisplatin-based anticancer therapy as a renoprotective adjuvant. | Hanioka et al., 2020 |
| Wogonin shows anti-inflammatory activity by inhibiting hyperpermeability, expression of CAMs, and also adhesion and migration of leukocytes, thereby endorsing its usefulness as a therapy for vascular inflammatory diseases. | Hong et al., 2020 |
| Wogonin reduces glycolysis and cell proliferation in cancer cells expressing wild-type p53 but not mutated p53. It also increases the expression of p53 and p53-inducible glycolysis and apoptosis regulator (TIGAR), while decreases glucose transporter 1 (GLUT1) and some key glycolytic enzymes. Expressing wild-type and mutant-type p53 in HCT116 p53-/-cells proved that the inhibitory outcome of wogonin on glycolysis in cancer cells is reliant on wild type p53. All these findings make clear the broad anti-tumor effect of wogonin and suggest a novel possibility for the therapeutic approach in cancer. The effect of wogonin on glucose uptake, lactate generation, and ATP content is assessing in hepatocellular, colon and ovarian cancer cells. | Huang et al., 2020 |
| Wogonin can ease liver fibrosis *via* regulating the activation and apoptosis of hepatic stellate cells, and possibly an efficient drug to treat and prevent liver fibrosis. Wogonin can largely increase cle-caspase3, cle-caspase9 expression and the ratio of Bax/Bcl-2 in T6 cells. | Jiang et al., 2019 |

EXCLI Journal 2020;19:635-640 – ISSN 1611-2156
Received: January 19, 2020, accepted: May 03, 2020, published: May 13, 2020

| **Key findings** | **Reference** |
|---|---|
| Wogonin shows its anti-inflammatory and chondroprotective potential by inducing mild oxidative stress throughout the generation of ROS and reduction of cellular GSH and modulating the cellular redox leading to the initiation of Nrf2/ARE pathways during activation of ROS/ERK/Nrf2/HO-1-SOD2-NQO1-GCLC signaling axis in OA chondrocytes. | Jiao et al., 2019 |
| Roles of wogonin in metabolism associated enzymes in human gastric cancer and lung adenocarcinoma cells show its various antitumor mechanisms. Various metabolic regulatory mechanisms shown by wogonin in different tumor tissues should be considered in antitumor therapy. With a combination of wogonin and certain enzyme inhibitors in energy, metabolism may stop the supply of energy to tumor cells and therefore helps in inhibition of tumor cell proliferation. Wogonin affects the energy metabolism and the acidic microenvironment in SGC-7901 cells by decreasing HIF-1α and MCT-4 expressions. In A549 cells, wogonin has no significant property on the energy metabolism, representing that the strong inhibitory effect of wogonin on cell proliferation may be induced by another mechanism, but not by the inhibition of the energy metabolism. | Khan and Kamal, 2019a |
| Drug interactions between wogonin and docetaxel in rats with mammary tumors indicated a linear pharmacokinetic profile of wogonin after oral administration. Additionally, the combined use of wogonin and docetaxel resulted in a major increase in both of their *in vivo* exposure, this may increase not only therapeutic but also toxic effects. As a result, the alterations of pharmacokinetics should be taken into concern when wogonin and docetaxel are co-administered. | Khan and Kamal, 2019b |
| Wogonin is used as a therapeutic agent for treating AR (Allergic Rhinitis), it shows the pharmacological activity by decreasing the infiltration of eosinophils and levels of T-helper type 2 cytokines. | Khushdil et al., 2019 |
| Wogonin shows a potent anti-influenza activity mediated by regulation of AMPK activation, signifying that wogonin has the potential to be developed as an anti-influenza drug. In the comparison of wogonin treatment after influenza, infection led to the up-regulation of interferon (IFN)-induced antiviral signal. Besides this, influenza virus infection in A549 cells induced 5' adenosine monophosphate-activated protein kinase (AMPK), phosphorylation and activation in a time-dependent approach and wogonin treatment led to the suppression of phosphorylation. | Kim et al., 2019 |
| Wogonin may reduce melanin synthesis by declining tyrosinase activity via the ER-ERK pathway. Western blot analysis also discovered that wogonin significantly inhibits the protein expression levels of TYR and JNK in A375 cells, which was inverted by ICI182780. Diminishing the protein expression levels of TYR and JNK may be associated with the regulation of melanin synthesis. RT-PCR shows that wogonin extensively inhibited mRNA expression levels of TYR, TRP-1, ERK2, JNK2, TRP-2 and ERK1, which may be concerned in the regulation of melanin synthesis. | Kong et al., 2019 |
| Wogonin repairs the lipotoxicity-induced decline of peroxisome proliferator-activated receptor α (PPARα) and adiponectin receptor 2 (AdipoR2) in hepatocytes, both *in vivo* and *in vitro*. Reduction of PPARα abolishes the defensive effect of wogonin on NCTC 1469 cells, also including the up-regulation of AdipoR2. It shows that wogonin may be a potential therapeutic agent for NAFLD via the up-regulation of hepatic PPARα/AdipoR2. | Liang et al., 2019 |
| Wogonin decreases the appearance of TCF-1, TCF-3, and LEF-1 and reserves nuclear accumulation of b-catenin as well as the binding of b-catenin and TCF-1, TCF-3, or LEF-1. All this shows that wogonin might inhibit the expression of VEGF, which is an important factor regulated by b-catenin. By taking together, wogonin may act as a potent inhibitor of Wnt/b-catenin and influence vascular permeability, and also provides new therapeutics in certain diseases. | Liau et al., 2019 |

EXCLI Journal 2020;19:635-640 – ISSN 1611-2156
Received: January 19, 2020, accepted: May 03, 2020, published: May 13, 2020

| Key findings | Reference |
|---|---|
| The SDs (solid dispersions) of wogonin had an advanced solubility than the physical mixtures. Based on XRD (X-ray diffraction) and DSC (differential scanning calorimetry), wogonin was transformed from a crystalline morphology to an amorphous structure. The considerably increased solubility of SDs and the additional preparation of arginine solution could notably increase the bioavailability of wogonin. | Luo et al., 2019 |
| Wogonin inhibits the activation of the TLR4/NF-κB pathway, which results in the inflammatory response and also protects human retinal pigment epithelium cells by LPS-induced barrier dysfunction. Many studies have confirmed that wogonin acts as a strong inhibitor of several other kinases involved in signal transduction. | Oomen et al., 2020 |
| Wogonin suppressed hyperglycemia, improved cardiac function, and mitigated cardiac fibrosis in STZ-induced diabetic mice. It also attenuates diabetic-induced cardiomyocyte apoptosis and necrosis. Wogonin treatment shows the properties of anti-oxidative stress and anti-inflammation. Wogonin potentially eases hyperglycemia associated cardiomyocyte impairment through inhibiting inflammation and oxidative stress. | Tan et al., 2019 |
| Wogonin has neuroprotective effects on amyloid-$\beta$ peptides- (A$\beta$-) induced toxicity. By taking wogonin orally it enhances the performance and also activates the neurite outgrowth of (Alzheimer's disease) AD cells by increasing neurite length and complexity of Tet-On A$\beta$42-GFP SH-SY5Y neuroblastoma cells (AD cells). It shows that wogonin might be a promising multifunctional drug for AD. | Wang and Cui, 2019 |
| Wogonin decreases the restoration ability of CSC (cancer stem cells) by inhibiting the formation and sinking the size of spheres. Wogonin at a concentration of 40–80 µM successfully minimizes potential risk from CSC. Taken together, this confirmed a new approach of wogonin for increasing a possible therapy for osteosarcoma. | Wang et al., 2019a |
| Wogonin shows activity against the hepatitis B virus, HBV DNA levels are attenuated wogonin therapy. These effects have lately confirmed in HepG2.2.15 cell lines. At the same time, wogonin harms HBeAg secretion. Wogonin has an inhibitory outcome on the production of HBsAg. Wogonin also appears to be of advantage in the supervision of "nonalcoholic fatty liver disease". Wogonin exhibits several of hepatoprotective properties. | Wang et al., 2019b |
| TF (Tissue factor) promoter activity of Wognin has been seen by inhibition of ERK/Egr-1- and JNK/AP-1-mediated transactivation of leading to downregulation of TF expression and this activity is induced by inflammatory mediators. | Wang et al., 2019a |
| Wogonin shows anti-oxidant activity, which possibly shows anti-inflammatory, anticancer, and antiviral and neuroprotective actions. Anxiolytic activity of Wogonin suggests a new mechanism of action, which includes interaction with the benzodiazepine (BZD) binding site of the GABAA receptor. While the safety record of wogonin is extraordinary and huge literature about its pharmacological effects is available while it has not been used in Western medicine as a pure chemical. | Wang et al., 2020 |
| Wogonin can inhibit the growth of tumor cells, stimulate apoptosis, and restrain angiogenesis. The molecular mechanisms involve reactive oxygen species, $Ca^{2+}$, tumor necrosis factor-related apoptosis-inducing ligand, tumor necrosis factor-alpha, and NF-κB. Additionally, the synergistic effect of wogonin with 5-fluorouracil, etoposide, and adriamycin to develop chemotherapy and reverse drug resistance has also been established. | Xing et al., 2019a |
| Wogonin shows its anticancer activity by producing the major reduction in the G1 cell-cycle regulatory proteins cyclin D1, 4 along with overexpression of cell-cycle inhibitory proteins p27 and cyclin-dependent kinase 2. Besides, the flavone considerably diminished the phosphorylated level of protein kinase B (AKT), and maintenance of low-catenin expression level was dependent on glycogen synthase kinase 3 activation at Ser9 in glioblastoma cells. | Xing et al., 2019b |

Received: January 19, 2020, accepted: May 03, 2020, published: May 13, 2020

| Key findings | Reference |
|---|---|
| Clinical impact of wogonin on the management of ovarian cancer results in increased expressions of Bax and p53 and down-regulated protein levels of estrogen receptor alpha (ER-$\alpha$), VEGF, and Bcl-2. Wogonin augmented caspase-3 cleavage and induced apoptosis in A2780 cells. MPP, a specific ER-$\alpha$ inhibitor, notably enhanced antitumor effects of wogonin in A2780 cells. | Zhang et al., 2019 |

***Conflict of interest***

The authors declare no conflict of interest.

## Conflict of interest

The authors declare no conflict of interest.

## Figures and Tables

**Table 1:**
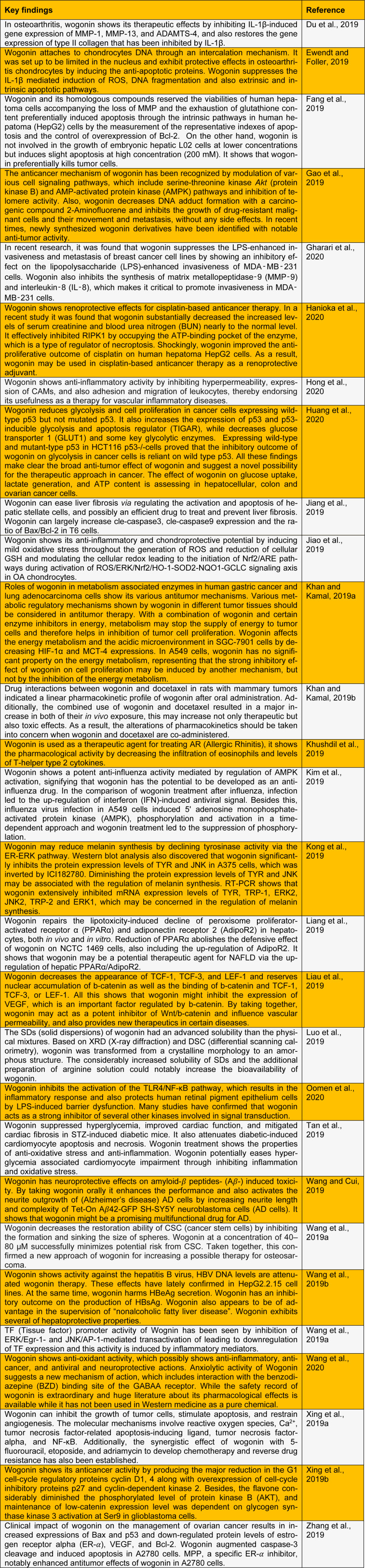
Current biological and pharmacological updates on wogonin
